# A Rare Case of Endocervical Adenocarcinoma of Gastric Type

**DOI:** 10.7759/cureus.33059

**Published:** 2022-12-28

**Authors:** Erinie Mekheal, Brooke E Kania, Ashima Kapoor, Vinod Kumar, Michael Maroules

**Affiliations:** 1 Internal Medicine, St. Joseph's Regional Medical Center, Paterson, USA; 2 Internal Medicine, St. Joseph's University Medical Center, Paterson, USA; 3 Hematology-Oncology, St. Joseph's Regional Medical Center, Paterson, USA; 4 Hematology-Oncology, St. Joseph's University Medical Center, Paterson, USA

**Keywords:** cervical malignancy, malignancy, post-menopausal bleeding, mucinous endocervical cancer, gastric endocervical adenocarcinoma, non-hpv cervical cancer

## Abstract

Gastric adenocarcinoma of the cervix (GAC) represents a rare mucinous endocervical cancer unrelated to human papillomavirus (HPV). GAC has been found to comprise approximately 10% of cervical adenocarcinomas internationally. As more cases have been identified, GAC has been further classified into subtypes such as poorly differentiated versus well-differentiated (also referred to as mucinous or adenoma malignum). This cancer coined the term “gastric” subtype due to its similarity to the pancreaticobiliary and gastric tissue lining. With limited data and similar histological and genetic features of GAC, this malignancy poses a challenge for clinicians when differentiating between metastasis from the gastrointestinal tract and GAC. Here, we present a case of a 55-year-old female who presented with postmenopausal bleeding and was found to have stage IA1 endocervical adenocarcinoma of gastric subtype. The purpose of this article is to introduce a rare type of gastric adenocarcinoma with a unique site of origin in order to better understand this disease process and potentially help clinicians better diagnose and treat patients with this malignancy in the future.

## Introduction

Endocervical adenocarcinoma (ECA) accounts for 20-25% of all cervical malignancies, and its frequency has been increasing in recent years, with the most common type identified as the "usual type" [1,2]. The second most common type is gastric-subtype adenocarcinoma (GAC), which accounts for 10% of the cases [1,2]. According to the most recent World Health Organization (WHO) classification of tumors of female reproductive organs, ECA is classified based on the morphologic features of the cells, the brisk mitotic activity, and the presence of eosinophilic cytoplasm [3,4]. In contrast to squamous cell carcinoma (SCC) of the cervix, which is most often caused by the human papillomavirus (HPV), approximately 10% of ECA in Western countries is independent of HPV [2]. Based on this data, a large cohort study of 409 invasive ECA was performed, which demonstrated there is a reliable separation of HPV-dependent ECA (HPVA) and non-HPV-dependent ECA (NHPVA) by morphology and a selective presence of immunohistochemistry panels (p16, p53, PR, vimentin) that further affect the biological and clinical behaviors of ECA, as well as the possible treatment options that can improve patient survival [2]. Due to the limited data surrounding this malignancy, patients may have been misdiagnosed, and currently, clear numbers for incidence are lacking [2]. Herein, we present a case of a 55-year-old female who presented with postmenopausal bleeding and was found to have stage IA1 endocervical adenocarcinoma of gastric subtype.

## Case presentation

This was a 55-year-old G7P5 female with a past medical history significant for diabetes mellitus, hypertension, and dyslipidemia and an addiction history significant for former tobacco use disorder (10 years), who presented with postmenopausal bleeding.

The patient was G7T5P0A2L5. She experienced menopause at age 45. The patient's family history was significant for a cervical cancer history in her maternal aunt.

**Figure 1:**
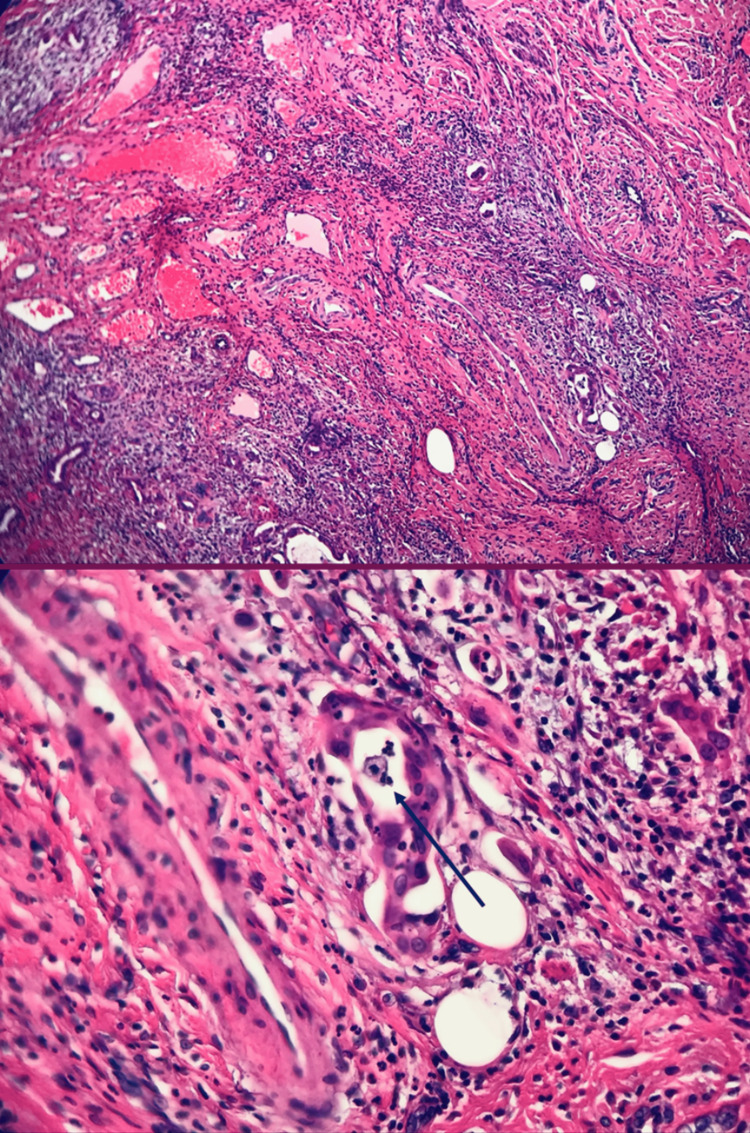
Focal endocervical adenocarcinoma in-situ of gastric type with invasive, mostly well-formed glands

The patient had an abnormal finding of atypical glandular cells during a Pap smear and colposcopy, which was unclear whether it was of endometrial or endocervical glandular origin. Subsequently, the patient was diagnosed with adenocarcinoma in situ-gastric type of the endocervix on cold knife conization biopsy. Three months following the cone biopsy, the patient underwent a total abdominal hysterectomy with bilateral salpingo-oophorectomy (TAHBSO). Post-surgical pathology was indicative of less than 0.4 cm tumor size. Pathology showed stromal invasion of less than 0.3 cm. Only three blocks of endocervix revealed invasive and focal in-situ HPV-independent adenocarcinoma-gastric type, with negative margins for invasive carcinoma. There was negative immunostaining for p63, which ruled out an adenosquamous carcinoma component. Focal lymphovascular invasion was identified and confirmed by immunostaining for D2-40, consistent with FIGO stage IA1 (Figure 1, Table 1).

**Table 1:** Histopathologic features following TAHBSO TAHBSO = total abdominal hysterectomy bilateral salpingo-oophorectomy

Histopathologic Findings		
Specimen	TAHBSO	
Tumor Site	9-12:00	
Tumor Size, Greatest Dimension in Centimeter	<0.4 cm	
Histologic Type	Adenocarcinoma HPV-independent, gastric-type	
Histologic Grade	Mostly G2, moderately differentiated	
Stroma Invasion	Present	
Depth of Invasion in Centimeter	<0.3 cm	
Lymphatic Vascular Invasion	Present (confirmed by immunostaining for D2-40)	
Margins	All margins negative for invasive carcinoma	
Margins for Adenocarcinoma In-Situ (AIS)	All margins negative for AIS	
Regional Lymph Nodes	No lymph nodes submitted or found	
Pathologic Stage Classification	pT1a1NxMx	
FIGO Stage	IA1	
Additional Findings	Endocervical adenocarcinoma in-situ	
Special Studies	p16 is negative	
Other Findings		
Endometrium	Proliferative	
Myometrium	Leiomyomata and adenomyosis	
Serosa	Unremarkable	
Cervix	Leiomyoma, intramural	
Bilateral Fallopian Tubes and Ovaries	Mostly unremarkable	
IHC Analysis		
Antibody/Tests	Clone	Results
Pan CK	AE1/3&PCK26	Highlights malignant glands
p63	4A4	Negative in malignant cells
p16	16P04	Negative
D2-40	Podoplanin	Highlights lymphovascular invasion

Following the procedure, the patient was evaluated by the Hematology-Oncology team, and the decision was made for Radiation Oncology referral for further management, who recommended adjuvant radiation therapy to the pelvis.

## Discussion

The International Criteria and Classification of Cervical Carcinoma (IECC) classified ECAs as HPV adenocarcinoma (HPVA) and non-HPV adenocarcinoma (NHPVA) based on the presence or absence of HPV-associated features of a severe pleomorphic nucleus with apical mitoses and apoptotic bodies (Table 2) [4].

**Table 2:** International Endocervical Adenocarcinoma Criteria and Classification (IECC) as well as World Health Organization (WHO) classification systems Adapted from [4]

International Endocervical Adenocarcinoma (ECA) Criteria and Classification (IECC)		World Health Organization (WHO)
Main Categories	Morphologic Subcategories	
HPV-related ECA, HPV Adenocarcinoma	Usual Type	Usual Type Adenocarcinoma
	Villoglandular Type	Villoglandular Adenocarcinoma
	Mucinous NOS Type	Mucinous Adenocarcinoma, NOS
	Mucinous Intestinal Type	Mucinous Adenocarcinoma, Intestinal Type
	Signet-Ring Cell Type	Mucinous Adenocarcinoma, Signet-Ring Type
	Stratified Mucin Producing Type	
Non-HPV Adenocarcinoma (Gastric Type)		Mucinous Adenocarcinoma, Gastric Type
Non-HPV Adenocarcinoma (Endometrioid Type)		Endometrioid Carcinoma
Non-HPV Adenocarcinoma (Clear-Cell Type)		Clear Cell Carcinoma
Non-HPV Adenocarcinoma (Mesonephric Type)		Mesonephric Carcinoma
Non-HPV Adenocarcinoma (Serous Type)		Serous Carcinoma
Invasive Adenocarcinoma, NOS		Invasive Adenocarcinoma, NOS

The term “gastric” subtype refers to a similar histopathological picture of gastrointestinal tissue lining [5]. Therefore, differential diagnoses include other subtypes of cervical adenocarcinoma or metastatic disease from other advanced-stage gastrointestinal, ovarian, or endometrial cancers, and unfortunately, there are limited immunohistochemistry markers that can be utilized to differentiate between these diseases [6].

GAC is the second most common ECA subtype, accounting for 10% of ECA patients with ages ranging from 29 to 76 years [2,7]. The most common initial presentation is abnormal vaginal bleeding, with or without vaginal discharge [7]. Despite being categorized as a subtype of mucinous ECA, GAC is not HPV-dependent, which has different etiologic, morphologic, immunohistochemical, and clinical features [2,5]. Mucinous minimal deviation adenocarcinoma (MDA)/adenoma malignum (AM) is a well-differentiated variant of GAC [5].

Cytologically, Kojima et al. have identified GAC as a mucinous adenocarcinoma with three main diagnostic features, including pale eosinophilic cytoplasm that is voluminous and cells presenting with distinct borders [8]. Additional findings associated with GAC include foamy glandular cells and dense eosinophilic cytoplasm [9]. GAC is histologically heterogeneous [9,10]. Glandular involvement varies in small-sized versus large cystic glands, dilated lumens appearing similar to Nabothian cysts, cysts with intraluminal papillary folds, or crowded tubular-appearing glands [9,10]. In contrast to NHPVA, GAC is usually concentrated in the upper cervical canal. Therefore, it manifests as a bulky cervix that spreads axially toward the lower aspect of the uterus and ectocervix compared with typical cervical adenocarcinoma [8,11].

It is currently thought that GAC may develop from a series of preexisting lesions that begin with gastric metaplasia that progress to lobular endocrine glandular hyperplasia (LEGH), atypical LEGH, gastric adenocarcinoma in situ, and finally invasive gastric adenocarcinoma [12,13]. This theory was confirmed when LEGH showed a similar IHC profile of MDA/GAC [14]. In addition, several studies showed that the pancreatic-biliary area, ovary, and fallopian tube may also be involved in GAC. It was noted that GAC appears histologically similar to primary tumors arising from these sites, e.g. pancreaticobiliary adenocarcinoma, ovarian mucinous cystadenoma/borderline tumors, or mucinous metaplasia/neoplasia of fallopian tube [10,15,16].

This information leads us to an important question in clinical oncology, which is how to differentiate primary endocervical GAC from metastatic gastric adenocarcinoma to the cervix. Overall, there are limited data on the immunophenotypic features of NHPVA and GAC [13]. GAC/MDA tumors are usually negative for p16, which has a high correlation with HPV status [17]. Recently, Carleton et al. explored the immunological profile of 45 patients with GAC. This study mentioned the use of immunostains such as CK7, CEA, CDX2, PAX8, MUC6, p53, ER, and PR to help with the definitive diagnosis of such cancer [18]. Accordingly, 31% of patients have MUC6 positivity in GAC, which represents a higher percentage when compared to typical cervical adenocarcinoma [8]. In GAC, next-generation sequencing has elucidated p53 genetic mutations, with an IHC study reporting p53 staining (more commonly diffuse positivity, less commonly null pattern) in 41% of patients (19/46 cases) [6,18].

A few recent studies were able to identify that MDA and the pseudoneoplastic LEGH have intracytoplasmic mucin that reflects the production of neutral, gastric/pyloric-type phenotypic mucin. In contrast, the normal endocervical glands, other endocervical adenocarcinomas, as well as gastric mucus cells, usually produce an equal amount of neutral and acidic mucin [19]. An immunomarker for this subtype of mucin, called HIK1083, which highlights the specific type of mucin in these tumors, may aid in the definitive diagnosis and help identify the primary origin of the tumor. Further studies showed that approximately 75% of GAC patients have HIK1083 positivity, and its positivity correlates to a decreased 5-year disease-specific survival rate (P<0.005) [8].

Although the marker HIK1083 may be promising in differentiating future GAC cases, in most parts of the world, this test is not commercially available for use [20]. Given the diagnosis of GAC can be difficult as this subtype of cervical cancer does not involve HPV screening, research has demonstrated a negative hr-HPV DNA and negative p16INK4a in GAC patients, which may aid in diagnosis [8,18,21].

In terms of the prognosis of GAC, Kojima et al. demonstrated that GAC had poor outcomes compared to other ECAs [8]. Even though MDA is low-grade, it is as clinically aggressive as high-grade varieties of GAC without any significant differences in age, stage, disease distribution, or outcome between MDA and poorly differentiated GAC [17]. There are many factors that could contribute to these results. Mainly, most patients present with advanced-stage disease at the time of initial diagnosis of GAC, with 59% of patients presenting with stages II-IV, and 41% of patients presenting with stage I disease [22]. In one study, approximately 64% of patients were found to have a lymphovascular invasion, and 7% of patients were found to have involvement of lymph nodes [6].

As GAC has been found to be unrelated to HPV in most cases, this suggests patients who receive the HPV vaccine with the intention of preventing cervical cancer may not be protected from GAC [23]. Additionally, HPV can be detected via PCR on cervical tissue that is adjacent to tissue demonstrating histologic features of GAC, which can lead to a false-positive test, which may impact management [24]. To combat this, these scenarios can be further analyzed via laser-capture microdissection PCR to confirm HPV negativity within the cells of the tumor [24]. A study by the Sankai Group has confirmed that these aggressive, rare types of ECAs are chemoresistant [25]. Another large retrospective study showed that GAC disease-specific overall survival (DSOS) is significantly lower than HPV-associated endocervical adenocarcinoma (UEA). Five-to-ten-year survival data were calculated at all stages. The results demonstrated that a five-year DSOS for Stage I UEA was successful 96% of the time in comparison to 62% of the time for Stage I GAC. However, when MDA cases were excluded from the GAC group, there was no statistically significant difference in survival or recurrence rates between both groups [22].

In terms of chemotherapy for GAC patients, one study utilized therapeutic options such as paclitaxel/carboplatin, irinotecan, and Tegafur/Imeracil/Oteracil therapies, with the selection of agents dependent on cancer staging and primarily with treatment in the adjuvant setting [26]. However, for most patients found to have stage IA disease of GAC, per current National Comprehensive Cancer Network (NCCN) guidelines, chemotherapy is not the first-line recommendation [27]. For patients with stage IA1 disease with lymphovascular space invasion (LVSI), such as our patient), modified radical hysterectomy with pelvic lymphadenectomy is recommended as a non-fertility-sparing option [27]. For patients with negative nodes, margins, and parametrium, observation is recommended unless they meet Sedlis criteria category 1 (for pelvic radiation) or 2B (for radiation and concomitant chemotherapy with either cisplatin or carboplatin) [27,28]. Patients who demonstrate positive pelvic nodes are recommended to undergo imaging for metastatic disease and if negative, to undergo external beam radiation therapy (EBRT) with concurrent chemotherapy such as cisplatin with or without vaginal brachytherapy [27].

Our patient underwent TAHBSO; however, she was unable to undergo pelvic lymphadenectomy and therefore her lymph nodes were not evaluated for evidence of disease. With this information unanswered, the radiation oncologist could not determine whether the patient had lymph node involvement and whether she was indicated for EBRT. A positron emission tomography (PET) scan was performed, which was negative for metastatic disease. Through interdisciplinary discussion, the decision was made to proceed with adjuvant EBRT given +LVSI with the presumption of greater than 0.3 cm of stromal invasion (2/3 Sedlis criteria). The patient was counseled regarding a higher risk of recurrence given +LVSI.

## Conclusions

Endocervical carcinoma represents approximately 20-25% of cervical malignancies, which can be further categorized into GAC, comprising approximately 10% of these cases. Ultimately, HPV vaccinations globally have led to a decrease in carcinomas derived from HPV; however, patients can still develop GAC, as this subtype is not derived from HPV. As the incidence of HPV ECA decreases, clinicians should have a high clinical suspicion of GAC for future patients. Additionally, clinicians who have utilized p16 as a marker for malignancy vs benign process may miss the diagnosis of GAC. The objective of our case report is to bring forth awareness of this rare malignancy especially considering its aggressive nature and diagnostic hurdles. The increased awareness of this disease may lead to future diagnostic tools that broaden our differential of cervical cancer and hopefully allow for earlier diagnosis and treatment of patients who develop this rare malignancy.
